# A226 EFFECT OF PHYSICAL ACTIVITY AMONG INDIVIDUALS WITH QUIESCENT OR MILDLY ACTIVE INFLAMMATORY BOWEL DISEASE: A SYSTEMATIC REVIEW

**DOI:** 10.1093/jcag/gwac036.226

**Published:** 2023-03-07

**Authors:** B Oketola, O Akinrolie, S Webber, H Singh, N Askin, R Rabbani, A Abou-Setta

**Affiliations:** 1 Applied Health Sciences; 2 Physical Therapy; 3 Internal Medicine; 4 University of Manitoba, Winnipeg, Canada; 5 George & Fay Yee Center for Healthcare Innovation; 6 Community Health Sciences, University of Manitoba, Winnipeg, Canada

## Abstract

**Background:**

Individuals with quiescent inflammatory bowel diseases (IBD) can continue to have several symptoms. Physical activity (PA) can improve immunological response and psychological health.

**Purpose:**

We performed a systematic review of trials investigating the safety and efficacy of PA as an adjunct therapy to manage persistent symptoms in quiescent or mildly active IBD.

**Method:**

We searched for randomised controlled trials (RCTs) and nonrandomised non-controlled trials (non-RCTs) in eight databases, trial registries and conference proceedings, published in English between 2011 and 2021. We focused on trials in adults (>18 years) using any form of PA as an adjunct therapy to medical therapy. Outcomes of interest were health-related quality of life, fatigue, joint pain, abdominal pain, stress, anxiety, and depression.

**Result(s):**

From 10,862 retrieved citations, we identified seven RCTs and one non-RCT that met our inclusion criteria. All trials deemed PA safe for individuals with quiescent or mildly active IBD. Clinical heterogeneity was noted among the trials for all outcomes. Six RCTs utilized parallel-group design while one utilized a cross-over design. Seven trials provided partially or fully supervised PA interventions, and one provided no supervision. All trials used different types of PA, which varied between running, resistance training, yoga, and aerobic exercises. The trials used different parameters to measure PA intensity including Peak Power Output, Resistance Intensity Scale for Exercise, percentage of maximum heart rate, ability to talk while running. Heterogeneity was noted in the duration and frequency of PA.

**Conclusion(s):**

Even though PA is safe for individuals with quiescent or mildly active IBD, heterogeneity in available trials make it difficult to ascertain precise estimates of treatment effect. This also presents a challenge when determining the specific modes of PA that are beneficial for individuals with quiescent or mildly active IBD. This highlights the need for standardization of the definitions of parameters involved in PA in IBD research.

**Please acknowledge all funding agencies by checking the applicable boxes below:**

CAG

**Disclosure of Interest:**

None Declared

